# The association between type 1 diabetes mellitus and the risk of immunoglobulin A nephropathy: a Mendelian randomization study

**DOI:** 10.3389/fmed.2024.1429369

**Published:** 2024-12-18

**Authors:** Chun-Hua Zhang, Yang Shen, Su-Mei Zhao

**Affiliations:** Department of Nephrology, Beijing Chaoyang Hospital, Capital Medical University, Beijing, China

**Keywords:** type 1 diabetes mellitus, IgA nephropathy, Mendelian randomization, single nucleotide polymorphisms, genome-wide association studies

## Abstract

**Objective:**

To investigate the potential causal relationship between type 1 diabetes mellitus (T1DM) and IgA nephropathy (IgAN) to deepen understanding of the association between these two conditions and to provide a scientific basis for future preventive and therapeutic strategies.

**Methods:**

This study employed Mendelian randomization (MR) analysis, using single nucleotide polymorphisms (SNPs) derived from genome-wide association studies (GWAS) as genetic instrumental variables (IVs), to assess the association between T1DM and IgAN. The analytical approaches included univariable and multivariable MR, along with sensitivity analyses such as Mendelian randomization-Egger (MR-Egger) and Mendelian Randomization Pleiotropy RESidual Sum and Outlier (MR-PRESSO), to evaluate the impact of heterogeneity and pleiotropy.

**Results:**

Univariable MR analysis using the IVW method revealed an odds ratio (OR) of 1.009 [95% confidence interval (CI): 1.032–1.206] for the association between T1DM and IgAN. Adjusted results from multivariable MR analysis indicated a significant relationship between T1DM and increased risk of IgAN; for example, after adjusting for triglycerides (TG), the OR was 1.534 (CI: 1.213–1.543). After adjustment for HOMA-IR, the OR was 1.303 (CI: 1.149–1.198). Sensitivity analyses, including MR-Egger regression intercept testing (*p* = 0.476), suggested no pleiotropy, and MR-PRESSO did not detect any influence from outlier SNPs.

**Conclusion:**

The findings suggest that T1DM is a factor in increasing the risk of IgAN, enhancing our understanding of the potential relationship between T1DM and IgAN and providing possible biological pathways for future disease prevention and intervention.

## Introduction

Type 1 diabetes mellitus (T1DM), also known as autoimmune diabetes, is characterized by permanent damage to pancreatic β-cells (1, 2). Studies have confirmed that T1DM is a key cause of diabetic nephropathy (DN) (3). Precise and early recognition of non-diabetic nephropathy (NDRD) has recently become increasingly important in medical practice. IgA nephropathy, also known as IgAN, is a chronic kidney disease characterized by the deposition of immunoglobulin A (IgA) in the glomeruli of the kidneys (4, 5), which may lead to a gradual decline in kidney function and potentially progress to end-stage renal disease. The incidence of IgAN exhibits noticeable geographical and ethnic variations worldwide, with higher rates in certain regions of Asia and Europe. Additionally, the prevalence of IgAN continues to rise, with significant geographical disparities (6). Exploring the causes and risk factors of IgAN is of potential great value for the prevention and treatment of NDRD. Especially in areas with high incidence of IgAN, these findings may lead to seminal understanding and coping strategies. Despite both T1DM and IgAN being immune-mediated diseases, research on the direct association between them remains relatively limited. Some studies suggest that they may share certain abnormalities in immune regulation and genetic susceptibility (7), e.g., variations in some HLA genotypes are considered risk factors for both diseases. Additionally, the state of chronic inflammation may act as a bridge in the development of both diseases (8, 9). Available epidemiological studies provide evidence of a possible link between T1DM and IgAN (10).However, most of these studies were conducted after participants had already been diagnosed with T1DM or T2DM, and therefore the current study had a small sample size and was mostly focused on a single center. In order to accurately understand the exact link between T1DM and IgAN, more extensive studies are necessary to overcome the limitations of existing studies.

Mendelian randomization (MR) is a research method that assesses causal relationships between exposure factors and disease outcomes using genotype as an instrumental variable (11). This method boasts the advantage of reducing the impact of confounding factors as genotypes are randomly assigned early in life and are typically not influenced by postnatal factors (12). By analyzing genetic variations associated with T1DM and IgAN, researchers can determine whether a causal relationship or just a superficial association exists between the two diseases in a more accurate way.

MR-based exploration of the potential association between T1DM and IgAN not only provides an in-depth understanding of the pathophysiology of these diseases but also facilitates the formulation of possible intervention strategies in the future to improve patients’ health and quality of life. The findings of this study will provide new perspectives and possibilities for research and treatment of immune-mediated diseases.

In this study, investigators aim to uncover potential links between these seemingly unrelated diseases by precisely selecting genetic markers associated with T1DM and observing the relationship between these genetic markers and the risk of IgAN. We employed a two-sample Mendelian randomization approach, utilizing summary statistics from genome-wide association studies (GWAS) to investigate the causal relationship between T1DM and IgAN. This approach allows us to overcome some of the limitations of traditional observational studies and provide more robust evidence for the association between these two conditions.

## Methods

### Study design

**Figure 1:**
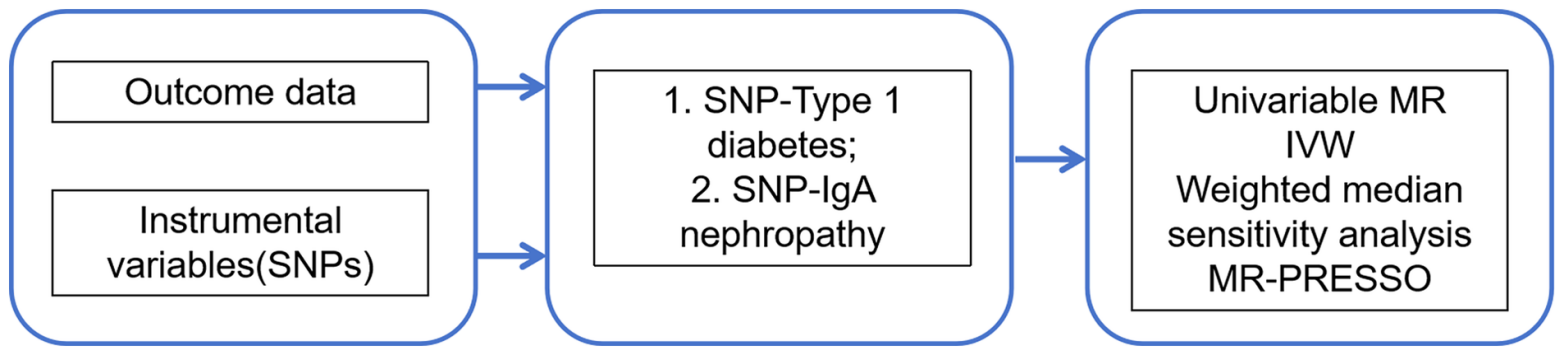
MR model of T1DM and risk factors for IgAN.

This study was conducted to assess the association between T1DM and IgAN using a conventional two-sample Mendelian randomization (MR) design, in which summary statistics from genome-wide association studies (GWAS) were retrieved, single nucleotide polymorphisms (SNPs) related to T1DM and IgAN were extracted as genetic instrumental variables, outcome GWAS summary statistics related to genetic variations in T1DM and IgAN from published large-scale GWAS meta-analyses and the FinnGen consortium were selected, and gene outcome associations were extracted for MR analysis, followed by corresponding sensitivity analyses (13). Figure 1 presents an overview of the design used in the current investigation. First, available genetic instrumental variables (IVs) were extracted from meta-analyses of T1DM. Second, IgAN was evaluated by collecting summary data for all SNPs from large-scale GWAS. Meanwhile, univariate two-sample MR and various sensitivity analyses were utilized to assess causal effects. Additionally, a Multivariate MR (MVMR) analysis was performed to control for confounding variables.

### Data sources

In this study, the latest and most comprehensive GWAS data were utilized, including 21,542 T1DM patients and 53,211 individuals of European descent as controls, and these data were obtained from 9 different cohorts. After performing uniform quality control on the data, researchers compared the genotypes with the TOPMed reference panel and conducted T1DM-related tests. Through meta-analysis, the research team integrated association data for 4,332,132 genetic variants and identified 60 SNPs with genome-wide statistical significance, including 40 known loci and 20 newly discovered loci, following strict linkage disequilibrium standards (LD *r*^2^ < 0.001, LD distance >10,000 kb). These 60 SNPs collectively explained 0.04 of the variance in T1DM. The selected IVs showed significant associations with T1DM, with the *F*-statistic >10. In non-European populations, summary statistics for T1DM were obtained by analyzing GWAS data from 213 T1DM patients of East Asian descent and 143,765 controls, in which a total of 16 SNPs were used as IVs, all exhibiting strong associations with T1DM (*p* < 5 × 10^−8^). In the meantime, population structure techniques were adopted to ensure the independence of SNPs, with those with minor allele frequencies (MAF) <0.42 removed. Additionally, the Medical Research Council (MRC)/Kidney Research UK National DNA Bank established a sample repository for 5 common glomerular diseases, including IgAN, with diagnoses for all IgAN patients confirmed through direct review of kidney biopsy reports and clinical records, while individuals with liver disease or Henoch–Schönlein purpura were excluded from the study. A GWAS meta-analysis of approximately 5957 European populations revealed genetic associations for IgAN. The study covered 977 IgAN patients from the MRC/Kidney Research UK National DNA Bank, as well as 4980 healthy controls from the 1958 British Birth Cohort and the UK National Blood Service. Because there was less than 1% sample overlap between the exposure and outcome data used in the study, samples can be considered relatively independent from each other (14). In addition, since all data were derived from previously published public studies and no new data collection was involved in this study, no additional ethical approval or participant consent was required.

### Univariate and multivariate MR analyses

In this study, Cochran’s *Q* tests were performed to assess heterogeneity using the inverse-variance weighted (IVW) and MR-Egger methods, possibly indicating potential violations of modeling assumptions. Meanwhile, the impact of potential pleiotropy on the exposure-outcome relationship was evaluated through the intercept test of MR-Egger regression, where a *p*-value <0.05 indicates the presence of pleiotropy. In cases of significant heterogeneity or horizontal pleiotropy, the MR Pleiotropy Residual Sum and Outlier (MR-PRESSO) method was employed to remove outlier SNPs (15). In addition, a leave-one-out test was conducted to identify individual SNPs with significant independent effects on MR estimates. Due to potential pleiotropic bias resulting from the common shared genetic loci in the HLA region among autoimmune diseases, SNPs in the HLA complex region were excluded, followed by repeated MR analysis. Since previous clinical studies indicated a certain association between glycemic and lipid metabolism characteristics with T1DM and IgAN, the summary GWAS data of these metabolic traits were introduced to this study, with the MVMR method applied in assessing the direct effect of T1DM after taking into account these metabolic characteristics (16). All analyses were conducted using the “TwoSampleMR,” “MR-PRESSO,” and “MVMR” packages in R software (version 4.2.0). All reported *p*-values were based on two-tailed tests, with a statistical significance level of 5%.

### Statistical analysis

In the two-sample MR analysis, traditional MR analysis is based on 3 key assumptions. First, the ideal IVs should meet the following criteria: (1) a significant genetic association with T1DM (in this study, the genetic association *p*-value should be <5 × 10^−8^); (2) unrelated to potential confounders of T1DM and IgAN; (3) only influencing IgAN through T1DM. Specifically, 4 different statistical methods were employed in this study to assess the causal relationship between T1DM and IgAN, including inverse-variance weighted (IVW), MR-Egger, weighted median, and maximum likelihood, with IVW as the primary one due to being considered the most accurate in estimating causal effects. However, the MR-Egger method may lead to bias in estimates due to pleiotropic genetic factors (17, 18). Additionally, an overall causal estimate for each risk factor was obtained by combining Wald ratio estimates for each SNP through the IVW method. Meanwhile, a random-effects model would be utilized in cases of heterogeneity, while IVW estimates may be biased if the selected SNPs are not effective IVs.

Therefore, 4 additional analytical models were further employed to enhance the robustness of the results. (1) The weighted median (WM) method was utilized, which requires over 50% of the weight in the meta-analysis to come from valid SNPs. (2) The MR-Egger method was used to detect and correct for potential bias introduced by pleiotropy. (3) The weighted model-based estimation method was employed to reduce bias and lower the type I error rate in smaller sample sizes. (4) The simple model-based approach was adopted to group SNPs with similar effects to test for consistency among them. For sensitivity analyses, we conducted several tests. Cochran’s *Q* statistic was used to detect heterogeneity in these analyses. Heterogeneity was further assessed through an intercept test in MR-Egger regression. A phenome scanner was used to detect associations between genes and other diseases, thus excluding the influence of genetic pleiotropy. We also performed leave-one-out analysis to identify any single SNP driving the overall effect, and created funnel plots to visualize potential directional pleiotropy. Furthermore, the reverse MR analysis was also conducted using data from European and East Asian populations to assess the causal relationship between IgAN and T1DM, and SNPs with *p*-value <5 × 10^−6^ were selected as IVs for more comprehensive results (see Table 1).

**Table 1:** Power calculation results.

				Statistical power at the given odds ratio				
Exposure	Outcome	Sample size	Cases	OR OR = 0.8 OR 1.0 OR = 1.3 OR 1.5 OR = 1.50				
T1DM-1	IgAN	3,937	543	1.00	1.00	1.00	0.05	1.00
T1DM-2	IgAN	37,037	2.0	0.43	0.21	1.00	0.43	0.65

OR, odds ratio; T1DM, type 1 diabetes mellitus; IgAN, IgA nephropathy.

## Results

### SNP and characteristics of participants

Table 2 shows the characteristics of the populations included in the exposure and outcome GWAS data. In the primary analysis, 60 SNPs were selected as IVs for T1DM.

**Table 2:** Description of relevant studies included in the GWAS analysis.

	Consortium	Sample size	Population	GWAS ID	Release time
Outcome					
IgAN	GWAS	10,534,735	European	prot-a-1406	2018
Exposures					
Triglyceride	GWAS	11,590,399	European	ebi-a-GCST90093051	2021
LDL	NA	111,638	European	ebi-a-GCST90093049	2022
HDL	NA	115,056	European	ebi-a-GCST90093013	2022
Insulin	GWAS	3,937	European	ebi-a-GCST90026413	2021
HOMA-B	MAGIC	36,466	European	ebi-a-GCST90006890	2021
HOMA-IR	MAGIC	37,037	European	ieu-b-118	2021
HbAlc	GLGC	46,368	European	ieu-b-5075	2010

GWAS, genome-wide association study; IgAN, IgA nephropathy; LDL, low-density lipoprotein; HDL, high-density lipoprotein; HOMA-B, homeostatic model assessment of beta-cell function; HOMA-IR, homeostatic model assessment of insulin resistance; HbA1c, glycated hemoglobin; NA, not available.

### Association between T1DM and IgAN

#### Univariate and multivariate MR analyses

The primary univariate analysis found a significant causal relationship between T1DM and elevated risks of IgAN. All four MR methods showed a *p*-value <0.05, indicating statistical significance for causality. Specific findings were as follows: the IVW method indicated an odds ratio (OR) of 1.009 (CI: 1.032–1.206); the Weighted Median method suggested an OR of 1.213 (CI: 1.121–1.232); and the Maximum Likelihood method showed an OR of 1.263 (CI: 1.226–1.235) (see Table 3).

**Table 3:** The effect of T1DM on IgAN by univariate and multivariate two-sample MR estimates.

Method	OR (95% CI)	*p*-value	*Q*-statistics	*p*_h_	Egger intercept	*p*_intercept_
MR-Egger	1.263 (1.226, 1.235)	0.0032	32.76	0.321	0.021	0.476
Weighted median	1.213 (1.121, 1.232)	0.0021	65.87	0.213		
Inverse variance-weighted	1.009 (1.032, 1.206)	0.0054				
MR-PRESSO						
Triglyceride	1.543 (1.213, 1.543)	0.0024				
LDL	0.989 (0.970, 0.982)					
HDL	0.845 (0.657, 0.743)					
Insulin	1.231 (1.021, 1.432)	0.0021				
HOMA-B	1.225 (1.321, 1.721)	0.0054				
HOMA-IR	1.303 (1.149, 1.198)	0.0032				
HbAlc	1.021 (1.032, 1.079)	0.0087				

OR, odds ratio; CI, confidence interval; *p*_h_, *p*-value for heterogeneity; MR, Mendelian randomization; PRESSO, Pleiotropy RESidual Sum and Outlier; LDL, low-density lipoprotein; HDL, high-density lipoprotein; HOMA-B, homeostatic model assessment of beta-cell function; HOMA-IR, homeostatic model assessment of insulin resistance; HbA1c, glycated hemoglobin.

In multivariate MR (MVMR) analysis, the association between T1DM and elevated risks of IgAN remained statistically significant after adjusting for different metabolic indicators. For example, the adjusted OR was 1.543 (CI: 1.213–1.543) for triglycerides (TG), 1.231 (CI: 1.021–1.432) for fasting insulin (FI), 1.225 (CI: 1.321–1.721) for HOMA-B, 1.303 (CI: 1.149–1.198) for HOMA-IR, and 1.021 (CI: 1.032–1.079) for glycosylated hemoglobin (HbA1c), respectively (see Table 3). However, the analysis of low-density lipoprotein cholesterol (LDL) and high-density lipoprotein (HDL) exhibited no similar trend, with the OR of 0.989 (CI: 0.970–0.982) and 0.845 (CI: 0.657–0.743) for LDL and HDL, respectively. Additionally, the reverse MR analysis revealed no T1DM-associated genetic susceptibility to IgAN (see Table 4).

**Table 4:** Bidirectional MR analysis.

Bidirectional MR	OR (95% CI)	Method	*p*-value
IgAN-T1DM (European)	0.65 (0.76–0.83)	Inverse-variance weighted	0.432
IgAN-T1DM (Asian)	1.01 (0.99–1.02)	Inverse-variance weighted	0.213

MR, Mendelian randomization; OR, odds ratio; CI, confidence interval; IgAN, IgA nephropathy; T1DM, type 1 diabetes mellitus.

#### Sensitivity analyses

Several sensitivity analyses were performed to further investigate the potential pleiotropy between exposure and outcome. Meanwhile, the heterogeneity of IVs was quantified using Cochran’s *Q* tests, where a *p*-value <0.05 indicated significant heterogeneity, following which a series of analyses were conducted to assess the presence of heterogeneity and horizontal pleiotropy. However, neither Cochran’s *Q* tests nor MR-Egger regression demonstrated evidence of heterogeneity or horizontal pleiotropy in the primary analysis (see Table 5).

**Table 5:** Primary MR results on IgAN.

Exposure	Method	OR (95% CI)	*p*-value
Triglyceride	IVW	2.072 (1.885, 2.279)	0.0032
LDL	IVW	0.954 (0.849, 0.998)	0.321
HDL	IVW	0.996 (0.920, 1.079)	0.431
Insulin	IVW	1.225 (0.871, 1.721)	0.0012
HOMA-B	IVW	0.760 (0.665, 0.868)	0.0012
HOMA-IR	IVW	0.989 (0.470, 2.079)	0.976
HbAlc	IVW	1.863 (1.626, 2.135)	0.003

MR, Mendelian randomization; IgAN, IgA nephropathy; OR, odds ratio; CI, confidence interval; IVW, inverse variance weighted; LDL, low-density lipoprotein; HDL, high-density lipoprotein; HOMA-B, homeostatic model assessment of beta-cell function; HOMA-IR, homeostatic model assessment of insulin resistance; HbA1c, glycated hemoglobin.

**Figure 2:**
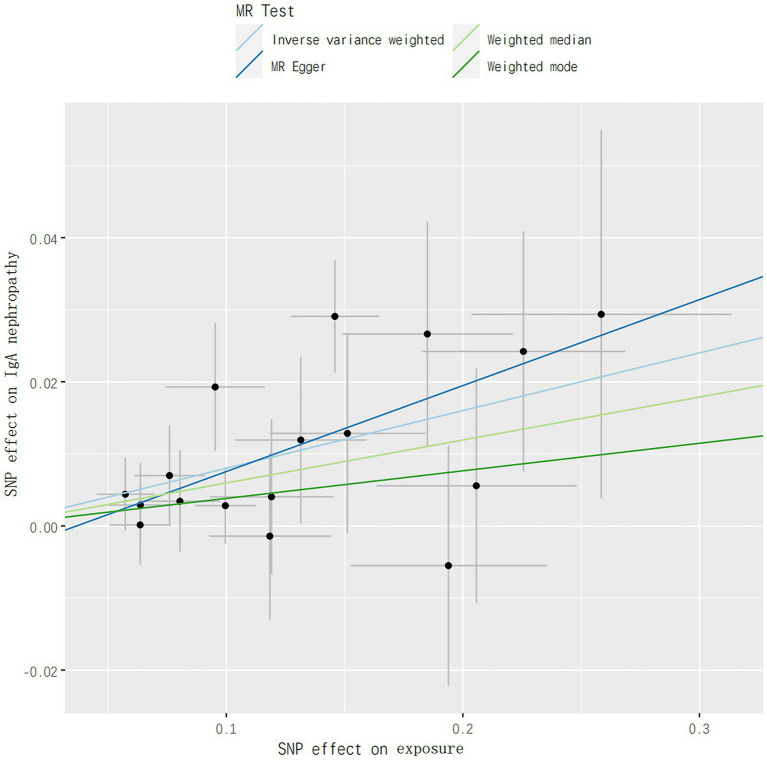
Genetically predicted association between T1DM and risks of IgAN. IVW, inverse-variance weighted; PRESSO, Pleiotropy RESidual Sum and Outlier; OR, odds ratio; CI, confidence interval; GWAS, genome-wide association studies.

**Figure 3:**
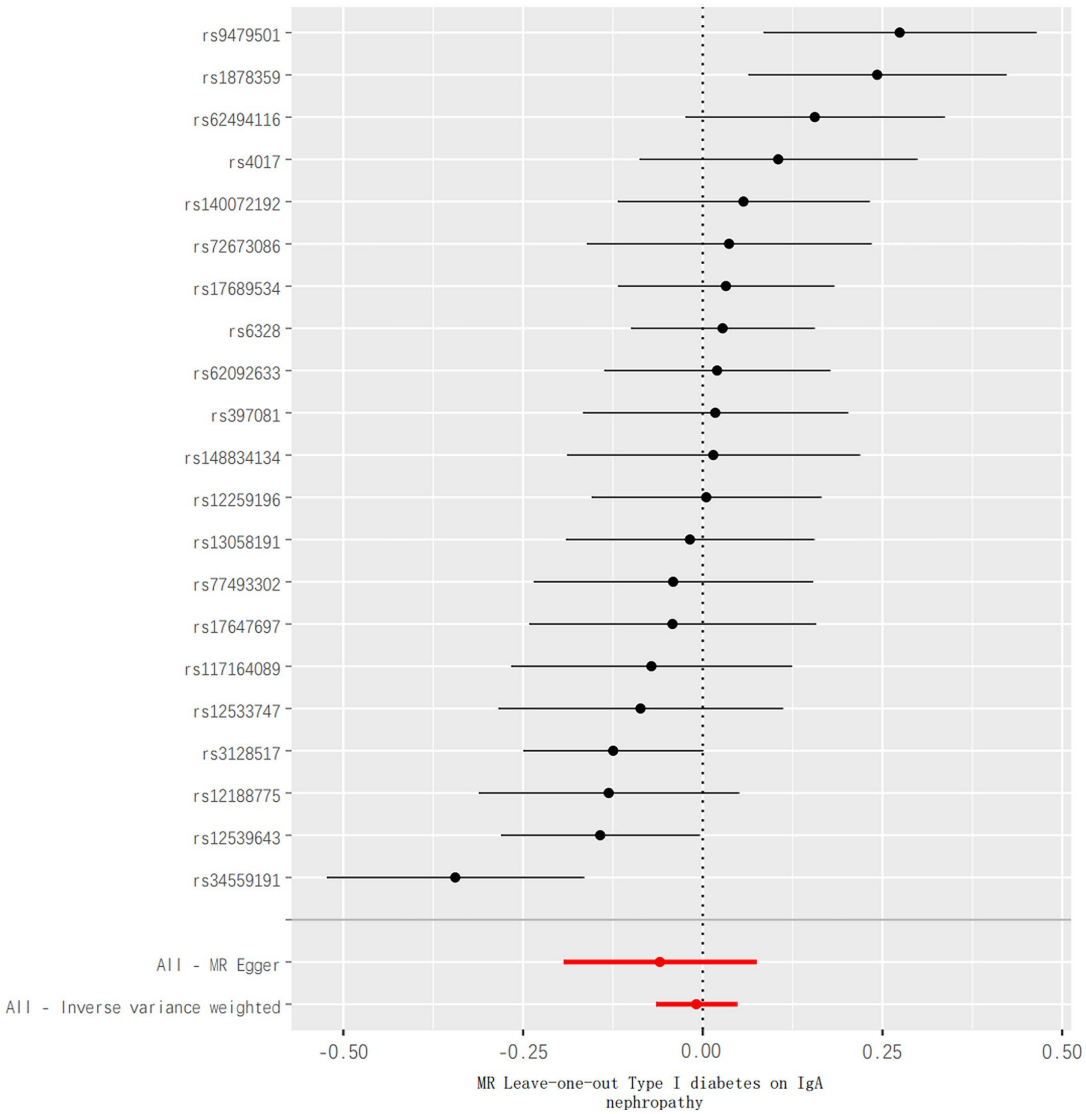
Leave-one-out analysis of the effect of TIDM on risks of IgAN. GWAS, genome-wide association studies.

**Figure 4:**
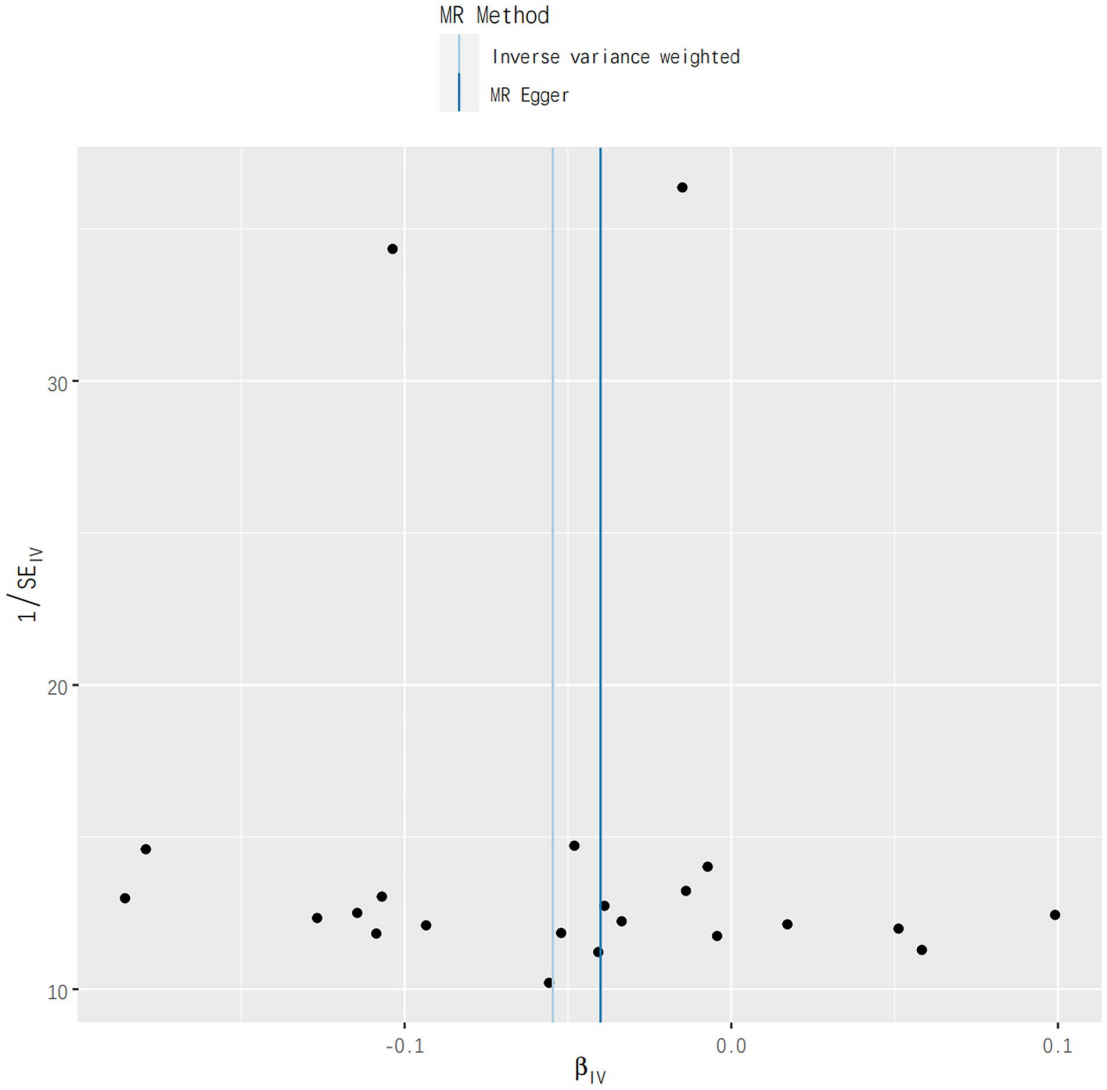
Funnel plot analysis results for the effect of genetically determined TIDM on risks of IgAN.

In addition, no aberrant SNPs were identified in the MR-PRESSO results. Therefore, 4 different methods were utilized to assess the results of the MR analysis, with scatterplots created accordingly (see Figure 2). In the leave-one-out analysis, no single SNP alone was found to drive the overall effect of T1DM on IgAN (see Figure 3). Figure 4 presents a relatively symmetrical distribution of IgAN variant effects, indicating no directional pleiotropy.

## Discussion

### Overview of main findings

This study provides genetic evidence through Mendelian randomization analysis that T1DM increases the risk of IgAN, indicating a potential causal relationship between the two. The findings suggest that T1DM is a factor in increasing the risk of IgAN, enhancing our understanding of the potential relationship between these diseases and providing possible biological pathways for future disease prevention and intervention.

Many diabetic patients suffer from IgAN,because renal biopsy is not universal, IgAN is sometimes misdiagnosed as DN, limiting the understanding of the link between diabetes and other kidney diseases (19).

### Methodological approach and results analysis

In this study, large-scale GWAS data were utilized to investigate the impact of genetically predicted T1DM on the risk of IgAN, and evidence supporting a causal effect of T1DM on IgAN was provided within the Mendelian randomization (MR) framework, with results showing an OR of 1.009 (CI: 1.032–1.206). Our bidirectional MR analysis revealed interesting results. While the forward MR analysis demonstrated a significant effect of T1DM on IgAN risk, the reverse MR analysis, using IgAN-associated SNPs as instrumental variables, did not show evidence of IgAN causing T1DM. This suggests that the relationship between T1DM and IgAN may be unidirectional. Even after adjusting for metabolic characteristics such as triglycerides, the association between T1DM and IgAN remained significant, which emphasizes the significant impact of T1DM on the risk of IgAN, even after taking into account the influence of metabolic factors.

Similarly, the adjusted OR of 1.303 (CI: 1.149–1.198) for HOMA-IR further confirmed the contribution of insulin resistance in T1DM to the risk of IgAN. Additionally, the intercept test of MR-Egger regression with a *p*-value of 0.476 indicated no pleiotropy in the analysis, which enhanced the credibility of the study findings, suggesting no bias resulting from selected SNPs as IVs due to genetic heterogeneity when estimating the impact of T1DM on the risk of IgAN. Moreover, MR-PRESSO identified no outlier SNPs, further confirming the robustness of the analysis results. Meanwhile, a similar phenomenon was observed in the research by Yan et al. (20), revealing that metabolic abnormalities in diabetic patients may exacerbate renal disease.

### Exploration of potential mechanisms

Although the results confirm a causal link between T1DM and IgAN, there is currently no evidence that IgAN is directly responsible for T1DM. This suggests that the link between the two may be through other undefined pathways. The prevalence of IgAN in T1DM patients is higher than expected, possibly related to other common autoimmune diseases in such patients, such as skin diseases and celiac disease, which involve pathogenic IgA antibodies. In addition, the existing research has revealed a close association between T1DM and IgAN with the human leukocyte antigen (HLA), and certain IgAN susceptibility loci are also associated with the risk of T1DM (21).

Given the complexity of the HLA region, more thorough sequencing may be beneficial for a more comprehensive understanding of the genetic background of autoimmune diseases. Currently, there is a lack of research exploring the risk profile of T1DM in IgAN patients, which provides research opportunities for future exploration of potential pathways, including drug treatments, inflammatory responses, and hormonal changes (22). Our multivariable MR analysis adjusted for potential comorbid confounding factors, indicating that LDL did not contribute to the causal relationship (23). Numerous clinical studies indicated that due to a high prevalence of lipid abnormalities in T1DM patients, adverse lipid profile, especially high LDL concentrations, may increase the risk of kidney disease (24). It has been shown that the decline of LDL antioxdant capacity is a characteristic feature of IgAN (24), Definitive mechanism to explain the potential link between LDL in T1DM and IgAN need further to clarify (25).

### Study strengths

(1) In this study, we investigated the possible link between T1DM and IgAN based on genetic data by applying two-sample MR, which has not been widely adopted in previous studies, thus providing a new perspective for research in this field. (2) In addition, this study integrates multiple MR analysis methods to scientifically enhance inferences about the causal relationship between exposure factors and disease outcomes. (3) To ensure the reliability of the findings, we also performed a series of sensitivity analyses using multiple complementary MR techniques designed to assess the stability of the observed associations and identify possible confounders, such as pleiotropic bias. With these approaches, we aim to provide a more solid evidence base to support further studies on the relationship between T1DM and IgAN. (4) It’s worth noting that we did not use the most recent large GWAS for IgAN by Kiryluk et al. (26), as our analysis was conducted before its publication. Future studies could benefit from using this larger cohort to further validate and refine our findings.

### Limitations

While providing genetic evidence of a potential causal relationship between T1DM and IgAN, the study also comes with some limitations. (1) The SNPs used as instrumental variables may not entirely meet all assumptions of Mendelian randomization, despite the robust results indicated by sensitivity analyses. (2) This study was primarily based on data from the European population, which may limit the generalizability of the results as genetic profiles and lifestyles in different ethnicities and geographical regions could influence the association between these two diseases. (3) While taking into account various potential confounding factors, the study may still overlook unknown or unmeasured confounders that could affect the interpretation of the results.

## Conclusion

In conclusion, this study provides genetic evidence that T1DM increases the risk of IgAN, indicating a potential causal relationship between the two. These findings emphasize the significance of monitoring and managing kidney health in T1DM patients in clinical practice to prevent and mitigate the progression of IgAN. Additionally, this study lays the foundation for further exploration of the specific biological mechanisms and potential therapeutic targets between these two diseases. However, given the limitations of the study, these findings need to be validated in a wider population in future research, along with an in-depth exploration of the molecular mechanisms and environmental factors regulating this association.

## Data Availability

The original contributions presented in the study are included in the article/supplementary material, further inquiries can be directed to the corresponding author.
